# A Murine Model of *Candida glabrata* Vaginitis Shows No Evidence of an Inflammatory Immunopathogenic Response

**DOI:** 10.1371/journal.pone.0147969

**Published:** 2016-01-25

**Authors:** Evelyn E. Nash, Brian M. Peters, Elizabeth A. Lilly, Mairi C. Noverr, Paul L. Fidel

**Affiliations:** 1 Department of Microbiology, Immunology, and Parasitology, School of Medicine, Louisiana State University Health Sciences Center, New Orleans, Louisiana, United States of America; 2 Department of Oral and Craniofacial Biology, Dental School, Louisiana State University Health Sciences Center, New Orleans, Louisiana, United States of America; 3 Prosthodontics, Dental School, Louisiana State University Health Sciences Center, New Orleans, Louisiana, United States of America; King's College London Dental Institute, UNITED KINGDOM

## Abstract

*Candida glabrata* is the second most common organism isolated from women with vulvovaginal candidiasis (VVC), particularly in women with uncontrolled diabetes mellitus. However, mechanisms involved in the pathogenesis of *C*. *glabrata*-associated VVC are unknown and have not been studied at any depth in animal models. The objective of this study was to evaluate host responses to infection following efforts to optimize a murine model of *C*. *glabrata* VVC. For this, various designs were evaluated for consistent experimental vaginal colonization (i.e., type 1 and type 2 diabetic mice, exogenous estrogen, varying inocula, and co-infection with *C*. *albicans*). Upon model optimization, vaginal fungal burden and polymorphonuclear neutrophil (PMN) recruitment were assessed longitudinally over 21 days post-inoculation, together with vaginal concentrations of IL-1β, S100A8 alarmin, lactate dehydrogenase (LDH), and *in vivo* biofilm formation. Consistent and sustained vaginal colonization with *C*. *glabrata* was achieved in estrogenized streptozotocin-induced type 1 diabetic mice. Vaginal PMN infiltration was consistently low, with IL-1β, S100A8, and LDH concentrations similar to uninoculated mice. Biofilm formation was not detected *in vivo*, and co-infection with *C*. *albicans* did not induce synergistic immunopathogenic effects. This data suggests that experimental vaginal colonization of *C*. *glabrata* is not associated with an inflammatory immunopathogenic response or biofilm formation.

## Introduction

Vulvovaginal candidiasis (VVC) is an opportunistic fungal infection caused by *Candida* species, in particular *C*. *albicans*, which affects 75% of healthy premenopausal women at least once [1]. An additional 5 to 10% of women suffer from recurrent VVC (RVVC), defined as 3 or more VVC episodes per year [2]. These infections present major quality-of-life issues in women worldwide, with signs and symptoms including itching, burning, discharge, and redness of the vulva and vaginal mucosa. Several exogenous factors, including use of high-estrogen oral contraceptives, hormone replacement therapy, antibiotic usage, immunosuppression, or uncontrolled diabetes are predisposing for VVC [1].

In the past decade, there has been a substantial paradigm shift in our understanding of the pathogenesis of *C*. *albicans* vaginitis. Rather than immune deficiencies defining susceptibility to infection, an acute inflammatory response mediated by polymorphonuclear neutrophils (PMNs) is strongly associated with the symptomatic condition [3]. An established mouse model of *C*. *albicans* VVC parallels the clinical condition and has been instrumental in identifying the requirements for immunopathogenesis (reviewed in [4]). Accordingly, vaginal epithelial cells are triggered by *C*. *albicans* to produce alarmins (S100A8) and pro-inflammatory cytokines (IL-1β) that promote the recruitment of PMNs to the vagina [5, 6]. *C*. *albicans* morphogenesis [6] and a putative sensitivity of the epithelial cells to the fungi are considered primary triggers of the response. The PMNs fail to function to reduce *C*. *albicans* burden while producing a strong acute inflammatory response [5, 6].

While *C*. *albicans* is the most common species isolated from VVC patients [7], *C*. *glabrata* is the second most common, with an incidence of 7 to 16% [8–10]. Furthermore, *C*. *glabrata* prevalence increases to 38% in VVC patients with uncontrolled diabetes mellitus, and is the predominant pathogen in women with type 2 diabetes [11, 12]. This high prevalence is intriguing given that *C*. *glabrata* does not undergo morphogenesis, which is considered a major virulence trait of *C*. *albicans* (reviewed in [13]) and genes that regulate the yeast to hyphae transition are required to trigger the immunopathogenic vaginal epithelial cell responses [6]. While *C*. *glabrata* is considered less virulent compared to *C*. *albicans*, it is innately resistant to azole antifungal drugs and displays higher resistance to all available azoles compared to most *C*. *albicans* isolates [14]. Successful treatment of *C*. *glabrata* VVC is thus challenging. Therefore, in depth studies, preferably in a robust animal model, are necessary to define the pathogenesis as well as to test new antifungal therapeutics.

Unfortunately, the *C*. *glabrata* mouse model of VVC (non-obese type 2 diabetic (NOD) mice), originally described by Fidel et al., was characterized by fairly inconsistent levels of *C*. *glabrata* colonization [15]. The lack of a fully reproducible model with consistent/sustained *C*. *glabrata* colonization has hindered the ability to adequately study pathogenesis or test therapeutic regimens. Therefore, the purpose of this study was to establish a reproducible murine model of *C*. *glabrata* vaginitis with consistent and appreciable levels of colonization to adequately investigate pathogenesis.

## Methods

### Ethics Statement

This study was carried out in accordance with the recommendations in the Guide for the Care and Use of Laboratory Animals of the National Institutes of Health. Mice were housed at LSU Health Sciences Center Animal Care facility. All animal protocols were reviewed and approved by the Institutional Animal Care and Use Committee (IACUC) of the LSU Health Sciences Center. All efforts were made to minimize pain and discomfort in the animals.

### Mice

Female C57BL/6 mice, 8–10 weeks old (Charles Rivers) were used for the majority of the study. For evaluation of a different mouse strain background, age-matched C3H/HeN mice were also tested. Nine to eleven week old KK.Cg-A^Y/J^ mice (Jackson Laboratories) were used as a model of type 2 diabetes.

### *Candida* isolates

*C*. *glabrata* clinical vaginal isolate BG2, also referred to as LF 574.92 was provided by Dr. Jack Sobel (Wayne State, Detroit, MI), and *C*. *albicans* strain DAY185, a prototrophic derivative of SC5314, was a gift from Dr. Aaron Mitchell (Carnegie Melon, Pittsburgh, PA). Isolates were grown overnight in yeast extract-peptone dextrose (YPD) broth, washed three times in sterile, endotoxin-free phosphate-buffered saline (PBS), counted on a hemocytometer, and diluted in PBS to the desired inocula.

### Type 2 diabetic murine model of *C*. *glabrata* vaginitis

KK.Cg-A^Y/J^ mice, which develop hyperglycemia, hyperinsulinemia, glucose intolerance and obesity by eight weeks of age, were injected subcutaneously with 0.1 mg of estrogen (β-estradiol 17-valerate; Sigma) dissolved in 0.1 ml sesame oil or sesame oil alone 72 hours prior to inoculation. Injections were administered weekly thereafter. Estrogen-treated mice were intravaginally inoculated by introducing 20 μl of PBS containing 2x10^6^—1x10^7^ *C*. *glabrata*.

### Type 1 diabetic murine model of *C*. *glabrata* vaginitis

To induce type 1 diabetes, mice were fasted for 4 h then injected intraperitoneally (i.p.) with two daily doses of 150 mg/kg of streptozotocin (STZ) (Sigma) freshly dissolved in sodium citrate buffer, pH 4.5 or buffer alone as a control. Mice were considered diabetic when blood glucose levels reached 200 mg/dL. Seventy-two hours prior to inoculation, diabetic mice (~75% of STZ-treated mice) or non-diabetic mice were injected subcutaneously with estrogen as described above. Estrogen-treated mice were intravaginally inoculated by introducing 20 μl of PBS containing 2x10^6^ or 1x10^7^ *C*. *glabrata*. In some experiments diabetic mice were also inoculated with 5x10^4^ *C*. *albicans* in PBS, or non-diabetic and diabetic mice were inoculated with 5x10^6^ *C*. *albicans* alone.

### Assessment of vaginal infections

Inoculated mice were evaluated longitudinally at 1, 3, 7, 14, and 21 days post-inoculation and euthanized on day 21 by CO_2_ asphyxiation followed by thoracotomy. Prior to vaginal lavages mice were anesthetized with isoflurane followed by gentle aspiration and agitation with 100 μl of sterile PBS. The lavage fluid collected was used to quantify fungal burden and PMN recruitment. The remaining fluid was centrifuged at 5000 rpm for 5 min to remove cellular debris, filtered through a 0.2-μm syringe filter, and stored at -80°C for future soluble mediator analyses. For quantification of fungal burden, a series of 10-fold serial dilutions was made using sterile PBS and plated onto CHROMagar^™^ plates. Colony forming units (CFUs) were enumerated after incubation at 35°C for 24 h and expressed as CFU/100 μl of lavage fluid. Lavage fluid (10 μl) was also smeared onto microscope slides, fixed with CytoPrep spray fixative (Fisher Sci), and stained using the Papanicolaou technique (“Pap-smear”). PMNs were identified by their size, staining appearance, and characteristic trinuclear lobes. For each smear (n = 4 to 8 mice/group), PMNs were manually counted in five nonadjacent fields by standard light microscopy using a 40X objective and averaged.

### Detection of S100A8 and IL-1β

Lavage fluids were analyzed for S100A8 or IL-1β protein using commercially available enzyme-linked immunosorbent assays (ELISA) and expressed as pg/ml (S100A8, R&D; IL-1β, eBioscience). For IL-1β pooled lavage fluid/group was required due to low concentrations.

### Lactate dehydrogenase activity assay

Lactate dehydrogenase (LDH) activity was measured in lavage fluid using the commercially available LDH Colorimetric Assay kit (ab102526; Abcam) and expressed as OD_450_.

### Biofilm Analysis

Vaginal tissue was excised 7 days post-inoculation from diabetic, estrogen-treated mice inoculated with *C*. *glabrata*, or non-diabetic, estrogen-treated mice inoculated with *C*. *albicans*, and bisected. Tissue was stained with 1 mg/ml Calcofluor White (Sigma) to visualize *C*. *albicans* yeast and hyphae, 50 μg/ml concanavalin A-Rhodamine conjugate (ConA-R) (Vector Laboratories) to stain *C*. *glabrata* yeast and extracellular matrix (ECM), and 2 μM of To-Pro-3 iodide (Life Technologies) to stain epithelial cell nuclei. Tissues were immersed in the stain mixture for 20 min followed by washing with PBS, and placed apical side up onto a glass microscope slides. The slides were covered with a glass coverslip and examined with an Olympus FluoView^™^ FV1000 confocal microscope and Fluoview software. Z-stack confocal images were taken at 1 μm intervals.

### PNA-FISH analysis of *C*. *glabrata-C*. *albicans* co-infection

Diabetic (STZ-treated), estrogen-treated C57BL/6 mice were co-inoculated with 1x10^7^ *C*. *glabrata* and 5x10^4^ *C*. *albicans* (DAY185) for 10 days prior to vaginal tissue excision. Tissues were fixed in formalin, paraffin embedded and sectioned onto slides by the Morphology Imaging Core (Louisiana State University Health Sciences Center). Cross-sections were stained by protein nucleic acid-fluorescent in situ hybridization (PNA-FISH) according to manufacturer’s protocol (AdvanDx) with fluorescein isothiocyanate (FITC)-labeled *C*. *albicans*/Cy3-labeled *C*. *glabrata* PNA probes. Fluorescence was captured with confocal microscopy using a 20X objective and 4X digital zoom.

### Statistics

Experiments were conducted using groups of 5–10 mice, or for type 1 diabetic mice, numbers depended on the percentage of mice that became hypergycemic after STZ injections in each experiment. Experiments used no less than 4 diabetic mice/group and were repeated, except where noted. All *in vitro* assays were repeated in duplicate, and the results averaged. Fungal burden and PMN quantification were analyzed using the Mann-Whitney U test, while the unpaired Student’s *t* test was used to analyze S100A8, IL-1β, and LDH data. Significant differences were defined at a confidence level where *P*<0.05. All statistical analyses were performed using Prism software (Graph Pad).

## Results

### Optimizing the *C*. *glabrata* vaginitis murine model

**Fig 1:**
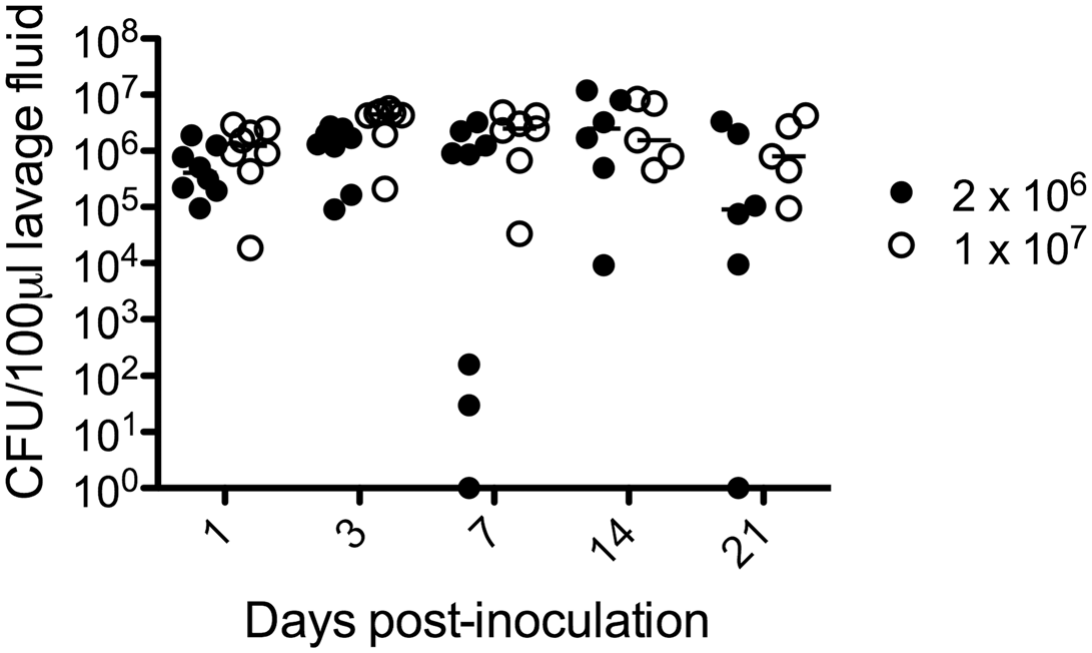
Effect of inocula size on vaginal fungal burden in type 1 diabetic estrogenized mice. Diabetes was induced in mice (n = 10/group) by two i.p. injections of STZ (150 mg/kg). Diabetic mice (n = 8/group) were estrogen treated (0.1 mg in sesame oil subcutaneously 72 h prior to inoculation) and inoculated with either 2x10^6^ (closed circle) or 1x10^7^ (open circle) CFUs of *C*. *glabrata* and followed longitudinally for 21 days to assess fungal burden in vaginal lavage fluids. Diabetic mice often succumb to uncontrolled diabetes that resulted in reduced numbers of mice/group for latter time points. Results are expressed as median CFU and are representative of no less than 3 experiments. Data were analyzed using the Mann-Whitney U test. Abbreviations: STZ, streptozotocin; CFU, colony forming units.

**Fig 2:**
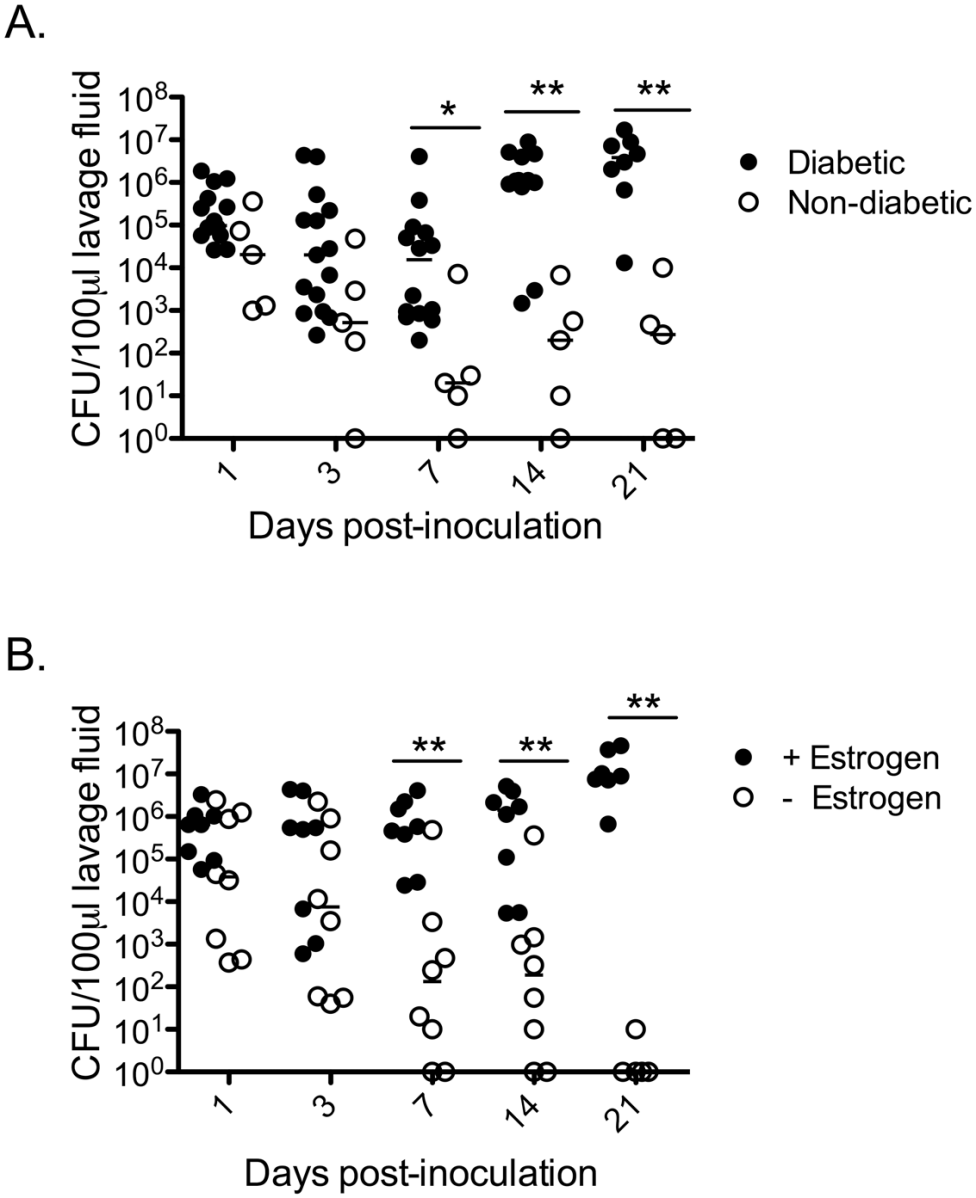
Effect of diabetic condition and estrogen treatment on fungal burden. To induce diabetes, mice were injected with STZ as described in Fig 1. STZ-treated diabetic (closed circle) (n = 7 or 8) or non-diabetic (open circle) (n = 2 or 3) mice were estrogenized (0.1 mg in sesame oil subcutaneously 72 h prior to inoculation) and inoculated with 1x10^7^ CFUs of *C*. *glabrata* **(A)**. STZ-treated diabetic mice (n = 4/group) were subcutaneously injected with 0.1 mg of estrogen in sesame oil (closed circle) or sesame oil alone (open circle) 72 h prior to inoculation with 1x10^7^ *C*. *glabrata* **(B)**. Fungal burden was assessed longitudinally on days 1, 3, 7, 14, and 21 in vaginal lavage fluids. Diabetic mice often succumb to uncontrolled diabetes that resulted in reduced numbers of mice/group for latter time points. Results are expressed as median CFU and are cumulative of two repeat experiments. Data were analyzed using the Mann-Whitney U test. Significance is denoted as *, *P*<0.05; **, *P*>0.01. Abbreviation: STZ, streptozotocin; CFU, colony forming unit.

Attempts to establish a consistent estrogen-dependent *C*. *glabrata* vaginitis mouse model included inoculating mice with varying inocula (2x10^6^-1x10^7^ CFUs) following various doses of STZ and injection schedules to induce a type 1 diabetic condition, or in type 2 diabetic mice (KK.Cg^A/J^) (similar to higher risk patients), co-inoculating with *C*. *albicans*, and supplementing the inocula with 10% glucose. Most strategies resulted in highly variable fungal burden or undetectable colonization. In contrast, two daily injections of STZ at 150 mg/kg resulted in a hyperglycemic rate of ~75% with low mortality and sustained high colonization levels in those diabetic mice after inoculation with 1x10^7^ *C*. *glabrata* CFU per estrogenized mouse and to a lesser extent with 2x10^6^ CFU per estrogenized mouse (Fig 1). The susceptibility of estrogenized type 1 diabetic mice to *C*. *glabrata* colonization prompted a formal assessment of the requirement for a diabetic condition and pseudoestrus. For assessment of the diabetic condition, both diabetic and non-diabetic STZ-treated mice were estrogenized and inoculated with *C*. *glabrata*. By day 7 post-inoculation, non-diabetic mice had significantly lower *C*. *glabrata* CFUs compared to diabetic estrogenized mice. By day 14 and 21 post-inoculation ~50% of the non-diabetic mice had no detectable *C*. *glabrata* (Fig 2A). For assessment of the pseudoestrus condition, diabetic mice were inoculated in the presence or absence of exogenous estrogen. By day 7 post-inoculation, fungal burden in non-estrogenized mice was significantly reduced and almost completely undetectable by day 21 compared to the high colonization through day 21 in estrogen-treated mice (Fig 2B).

**Fig 3:**
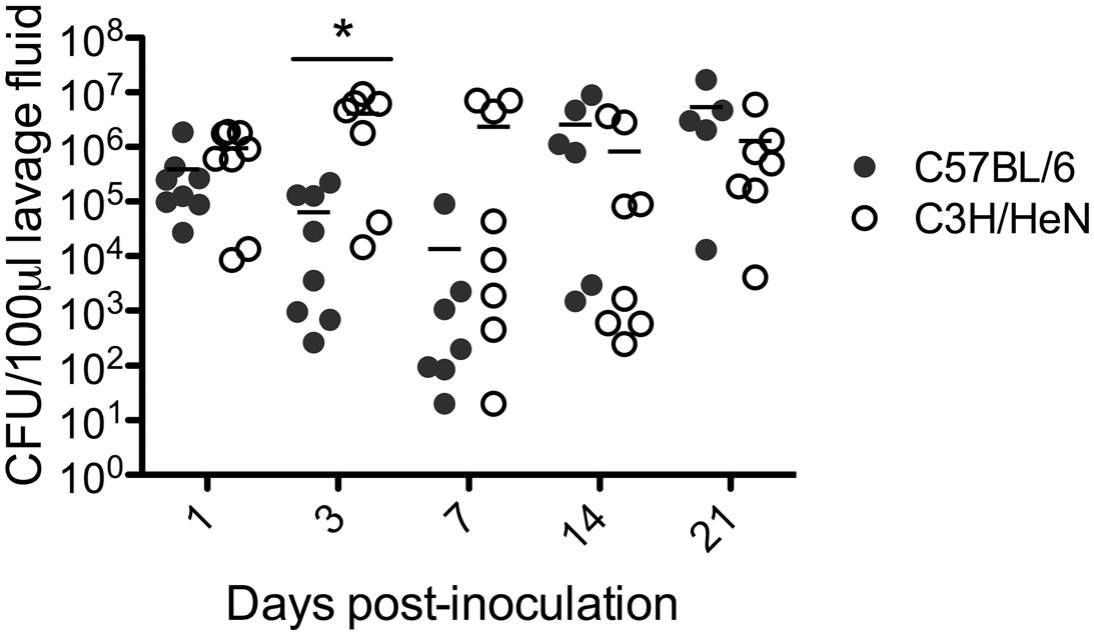
Effect of mouse strain on fungal burden in diabetic estrogenized mice. Diabetes was induced in C57BL/6 (H-2^b^) and C3H/HeN (H-2^k^) mice as described in Fig 1. STZ-treated diabetic mice (n = 8/group) were estrogenized and inoculated with *C*. *glabrata*. *C*. *glabrata* CFUs were quantified longitudinally in C57BL/6 (closed circle) and C3H/HeN (open circle) mice from vaginal lavage fluids collected on days 1, 3, 7, 14, and 21 post-inoculation. Diabetic mice often succumb to uncontrolled diabetes resulting in reduced mice/group in latter time points. Results are expressed as median CFU and only one experiment was performed. Data was analyzed using the Mann-Whitney U test. Significance is denoted as *, *P*<0.05. Abbreviation: STZ, streptozotocin; CFU, colony forming unit.

Several studies have reported no differences in susceptibility to experimental *C*. *albicans* vaginitis among various mouse strains [5, 16, 17]. To determine whether this was true as well for *C*. *glabrata* vaginitis, a study evaluating vaginal fungal burden in diabetic-estrogenized C3H/HeN (H-2^k^) mice in parallel with C57BL/6 (H-2^b^) mice was performed. Results showed that both strains harbored similar fungal burden throughout the 21-day period (Fig 3).

### *C*. *glabrata* vaginitis does not elicit immunopathology

**Fig 4:**
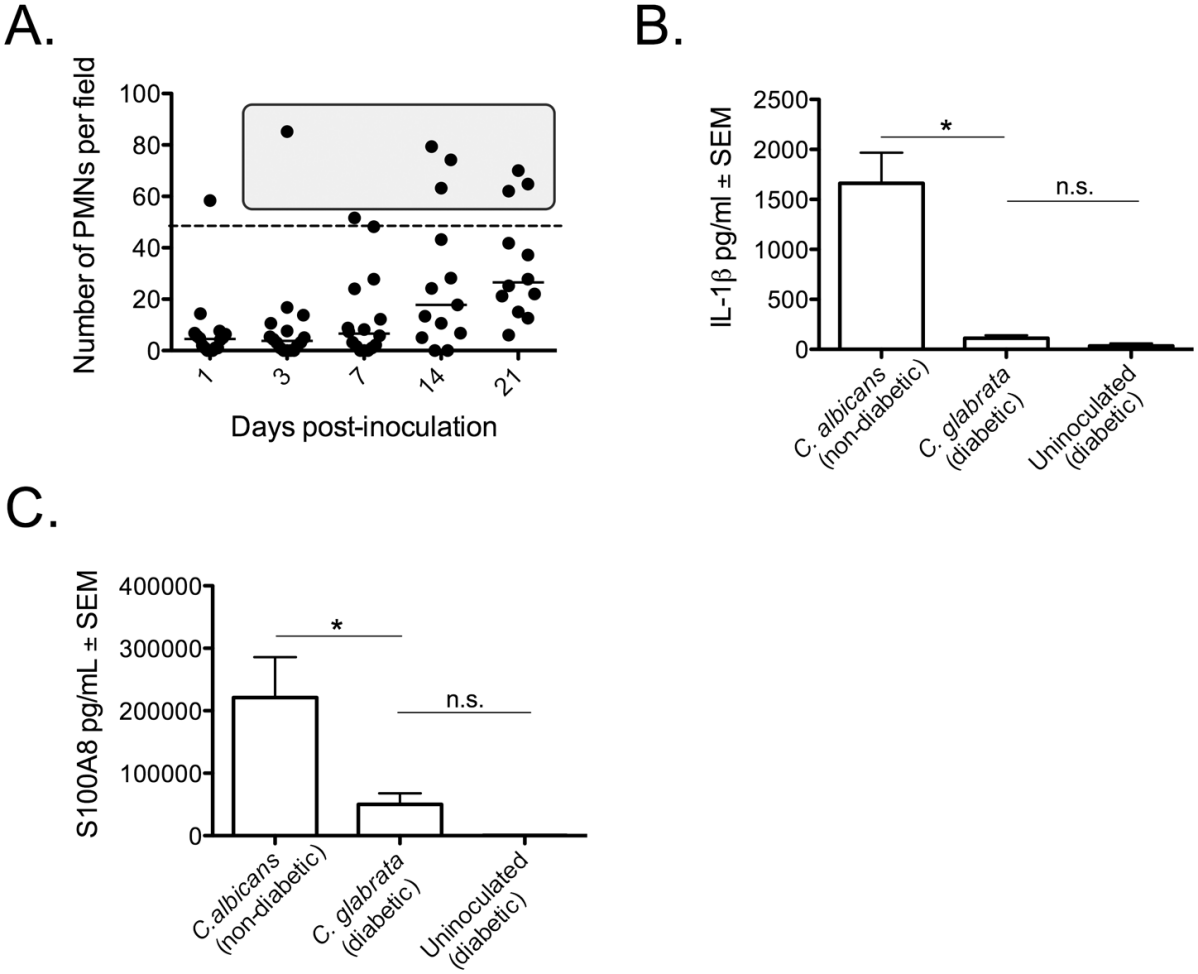
Lack of immunopathology during *C*. *glabrata* vaginitis. Diabetes was induced in mice as described in Fig 1. STZ-treated diabetic mice (n = 8) were estrogenized, inoculated with *C*. *glabrata*, and underwent vaginal lavage on days 1, 3, 7, 14, and 21 to assess PMN infiltration into the vaginal lumen (the dashed line denotes the high-responder cutoff, further described in [5], and the gray box is representative of typical PMN migration observed in 75% of *C*. *albicans* vaginal infections) **(A)**. Lavage fluids from day 7 post-inoculation were collected from *C*. *glabrata*-infected mice (diabetic, estrogenized, 1x10^7^ CFUs) and uninoculated mice (diabetic, estrogenized) and analyzed for IL-1β **(B)** and S100A8 **(C)** protein levels by ELISA. IL-1β and S100A8 were also evaluated in banked specimens from *C*. *albicans* inoculated (5x10^6^ CFUs) estrogenized non-diabetic mice on day 7 post-inoculation. The results are expressed as median PMN counts and mean protein concentration ± SEM and cumulative of two repeat experiments. For IL-1β quantification, pooled lavage fluids from *C*. *glabrata*-inoculated mice (n = 15), *C*. *albicans*-inoculated mice (n = 8), and uninoculated diabetic mice (n = 4) were analyzed. For S100A8 quantification, individual lavage fluids from *C*. *glabrata*-inoculated mice (n = 10), *C*. *albicans*-inoculated mice (n = 10), and uninoculated diabetic mice (n = 4) were analyzed. Data were analyzed using the unpaired Student’s *t* test. Significance is denoted as *, *P<*0.05; n.s., not significant. Abbreviations: STZ, streptozotocin; PMN, polymorphonuclear leukocyte; CFU, colony forming units; ELISA, enzyme-linked immunosorbent assay; SEM, standard error of the mean; IL-1β, interleukin-1β.

The immunopathology of *C*. *albicans* vaginitis is characterized by elevated vaginal PMN infiltration, and elevated levels of the inflammatory cytokine IL-1β, the alarmin S100A8, and LDH [5, 6]. This hallmark inflammatory response is not dependent on a strict threshold level of fungal burden, but requires morphogenesis [6]. Because *C*. *glabrata* does not form hyphae, we were interested in whether *C*. *glabrata* vaginal colonization resulted in any evidence of the immunopathologic response. Thus, PMN infiltration was quantified longitudinally in vaginal lavage fluid of estrogenized diabetic *C*. *glabrata*-inoculated mice, and S100A8 and IL1β were evaluated at day 7 post-inoculation for comparisons to fluids collected from uninoculated estrogenized diabetic mice, as well as to banked specimens from day 7 *C*. *albicans* infected non-diabetic mice. PMN infiltration in *C*. *glabrata* inoculated mice was low; only 20% of mice had PMN counts in the range that is typical of >75% of *C*. *albicans* infected mice [5, 6] (Fig 4A). Similar PMN levels were observed in inoculated C3H/HeN mice (data not shown). Concentrations of IL-1β and S100A8 alarmin in lavage fluids of *C*. *glabrata*-inoculated mice were not significantly different compared to that from uninoculated diabetic mice and significantly reduced compared to the much higher levels in both non-diabetic *C*. *albicans*-inoculated mice (standard VVC conditions) (Fig 4B and 4C) as well as diabetic *C*. *albicans*-inoculated mice (data not shown).

### *C*. *glabrata* does not form biofilm *in vivo*

**Fig 5:**
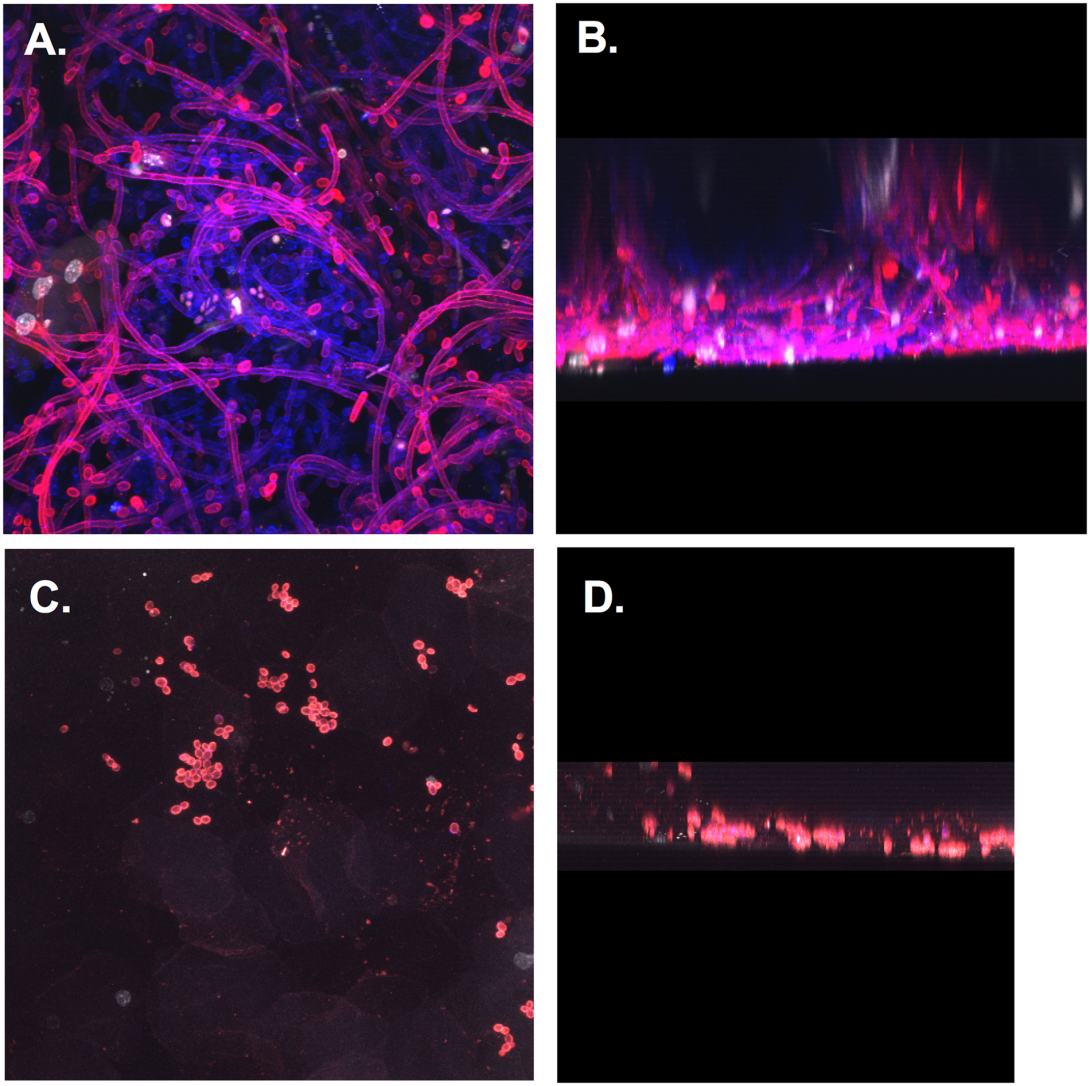
Lack of biofilm formation during *C*. *glabrata* vaginitis. Vaginal tissues from *C*. *glabrata*-inoculated mice (diabetic, estrogenized, 1x10^7^ CFUs) and *C*. *albicans*-inoculated mice (non-diabetic, estrogenized, 5x10^6^) were excised 7 days post-inoculation to assess *in vivo* biofilm formation. Vaginae were stained with calcofluor white (blue) to visualize *C*. *albicans* yeast and hyphae **(A)**, Con A-R to visualize *C*. *glabrata* yeast and ECM **(C)**, and To-Pro-3 iodide (white) to visualize epithelial cell nuclei. Z-stack images were rotated to visualize height of *C*. *albicans* **(B)** and *C*. *glabrata* **(D)** biofilm. Confocal images were captured using a 60X oil emersion objective. The figure shows representative images of areas of biofilm growth from two repeat experiments using groups of 3 mice each. Abbreviations: CFU, colony forming unit; ConA-R, Concavalin A-Rhodamine conjugate; ECM, extra-cellular matrix.

One shared trait of *C*. *glabrata* and *C*. *albicans* is their ability to form biofilm on abiotic surfaces, albeit with different characteristics. *C*. *albicans* biofilm architecture is well defined (reviewed in [18]) and requires the yeast-to-hyphal transition to create a complex three-dimensional structure. In contrast, *C*. *glabrata*, which does not undergo morphogenesis, forms a loosely adherent biofilm characterized by a dense network of yeast cells embedded in extracellular matrix [19]. Previous studies have demonstrated that *C*. *albicans* forms a robust biofilm *in vivo* during vaginal infection [20]. Therefore, it was important to determine whether *C*. *glabrata* could form a biofilm on vaginal mucosa. For these experiments vaginae excised from 7 day *C*. *glabrata*- or *C*. *albicans*-inoculated mice were evaluated for cross-sectional depth and cellular architecture by confocal microscopy. Results showed that *C*. *albicans* biofilm consisted of dense hyphal networks (Fig 5A) resulting in significant three-dimensional structure with typical biofilm architecture (Fig 5B), similar to what has been reported previously for mucosal biofilms [20]. Alternatively, *C*. *glabrata* colonization was sparse, forming only a monolayer of cells without any appreciable extracellular matrix (Fig 5C and 5D), which did not resemble biofilm.

### *C*. *glabrata-C*. *albicans* co-infections do not have synergistic effects on virulence

**Fig 6:**
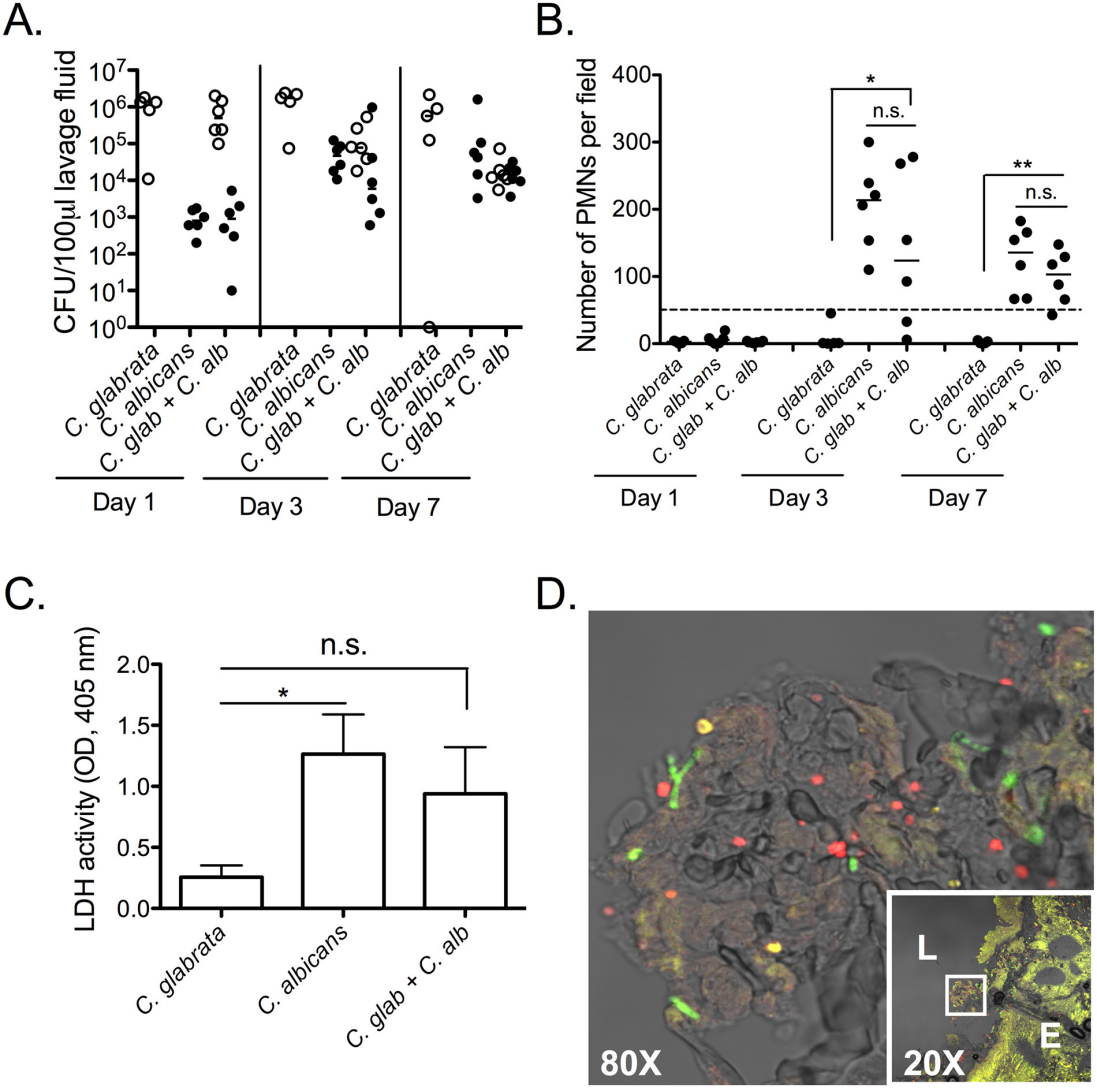
Lack of synergistic effects on virulence during *C*. *glabrata* and *C*. *albicans* co-infections. Diabetes was induced in C57BL/6 mice (n = 20) as described in Fig 1. STZ-treated diabetic mice were estrogenized and inoculated with 1x10^7^ *C*. *glabrata* (n = 5), 5x10^4^ *C*. *albicans* (n = 6), or both (n = 6). Mice were lavaged on days 1, 3, and 7 post-inoculation to measure fungal burden (*C*. *glabrata*, open circle; *C*. *albicans*, closed circle) **(A)** and PMN infiltration (dashed line denotes high-responder cutoff, further described in [5]) **(B)**. LDH was quantified in lavage fluids from day 7 post-inoculation **(C)**. Vaginal tissues were excised from co-inoculated mice on day 10, and *C*. *glabrata*-specific (red) and *C*. *albicans*-specific (green) PNA-FISH probes were used to stain fungal cells **(D)**. Fluorescent images of vaginal epithelium (E) and lumen (L) were captured using a 20X objective (insert) and 4X digital zoom (expanded insert 80X). Results are expressed as median CFU and PMN counts and mean LDH ± SEM. Shown are representative data from two experiments. Fungal burden and PMN data were analyzed using the Mann-Whitney U test, and LDH concentrations were analyzed with the unpaired Student’s *t* test. Significance is denoted as follows, *, *P*<0.05; **, *P*<0.01; n.s., not significant. Abbreviations: STZ, streptozotocin; LDH, lactate dehydrogenase; PNA-FISH, protein nucleic acid-fluorescent in situ hybridization; E, epithelium; L, lumen; SEM, standard error of the mean.

*C*. *glabrata* is often found as a co-infecting microbe with *C*. *albicans* during VVC. A recent study showed that 10.3% of VVC patients had mixed infections with the majority of these cases consisting of *C*. *albicans* and *C*. *glabrata* [21]. Therefore we assessed *C*. *glabrata-C*. *albicans* co-inoculated mice for synergistic responses. Using the type I diabetic mouse model, we inoculated mice with *C*. *glabrata* and sub-optimal levels of *C*. *albicans* (two logs lower than standard for SC5314 or SC5314 derivative DAY185 to allow for potential synergistic effects on responses). *C*. *glabrata* fungal burden was fairly constant over the 7 day period, with equivalent levels in mono- and co-inoculated mice. The exception was lower levels of fungal burden in co-inoculated mice at day 7. *C*. *albicans* fungal burden was also consistent between mono- and co-inoculated mice with similar increases over time in both settings (Fig 6A). At days 3 and 7 post-inoculation, PMN infiltration was high in co-inoculated mice, but this was not significantly different than what was observed in mice inoculated with *C*. *albicans alone* (Fig 6B). We also measured LDH in lavage fluid at day 7 post-inoculation as a marker of tissue damage. *C*. *albicans* mono- and co-inoculated mice had similar levels that were both significantly higher compared to that from mice inoculated with *C*. *glabrata* alone (Fig 6C). Lastly, PNA-FISH analysis using probes specific for *C*. *albicans* and *C*. *glabrata* revealed little to no interaction or co-localization of the two species on vaginal tissues in co-inoculated mice (Fig 6D).

## Discussion

*C*. *glabrata* VVC presents a challenge to clinicians as it is commonly diagnosed but difficult to manage adequately. Effective antifungal treatment does not always result in reduction in symptomatology calling into question whether *C*. *glabrata* is a causative agent in all cases [22]. With the increasing prevalence of *C*. *glabrata* infection, it is extremely important to utilize a reproducible and robust animal model whereby pathogenesis can be studied and therapeutic options evaluated. A previous model using NOD mice had inconsistent rates of colonization [23], and non-diabetic models failed to support *C*. *glabrata* colonization for any extended period of time [24]. Thus, our first objective was to attempt to establish conditions in mice that produced consistent and reproducible *C*. *glabrata* colonization could be achieved. Following several experimental designs (i.e., type 1 or type 2 diabetic conditions, varying inocula, ± pseudoestrus, supplementation of the inocula with glucose, and co-inoculating with *C*. *albicans*), we concluded that inoculum size was important and that pseudoestrus and a type 1 diabetic condition were strong requirements for consistent high levels of colonization. A high blood glucose level by 7 days post-STZ injection is a relatively short period to achieve the diabetic condition and a positive attribute of the model. The requirement for the diabetic condition in the model confirms an important link between diabetes and consistent *C*. *glabrata* colonization, although it remains unclear how the diabetic condition contributes to promoting colonization. It does not appear that local (vaginal) glucose is important as supplementing the inocula with glucose had no effect on promoting colonization in non-diabetic mice and less than 50% of type 1 diabetic mice had detectable glucose in vaginal secretions (data not shown).

Regarding colonization under optimized conditions, levels appear to be reduced by day 3 and 7 post-inoculation before increasing again at day 14 and 21. This delay could be the time necessary for *C*. *glabrata* to fully adapt to the environmental conditions *in vivo*. Adaptation factors may represent possible targets for treatment and windows for effective management of *C*. *glabrata* infections. Finally, there appears to be no effect of mouse strain on susceptibility to colonization, as two mouse strains with different haplotypes show similar levels of colonization under the type 1 diabetic condition, similar to what was previously observed with *C*. *albicans* in non-diabetic mice [5, 16, 17]. Of note, the type 2 diabetic mice, which failed to support consistent colonization, showed a peculiar property that may have affected the outcome. Interestingly, the hyperglycemic condition was lost under pseudoestrus (data not shown) that is required for colonization. Thus, a type 2 diabetic model of *C*. *glabrata* VVC is still possible if the diabetic condition can be maintained.

The immunopathogenic inflammatory response associated with *C*. *albicans* vaginitis is considered the underlying cause of symptomatology [3, 5]. The immunopathology (via LDH concentrations) is characterized by high S100A8 and IL-1β production [6]. In stark contrast, there is no evidence of immunopathology during *C*. *glabrata* vaginitis at least as it pertains to these specific markers. In fact, the response to *C*. *glabrata* infection is similar to the response observed with a yeast-locked mutant of *C*. *albicans*, which was characterized by a lack of immunopathology despite significant colonization [6]. Of note though, *C*. *albicans* mutants locked in the yeast morphology are capable of colonizing non-diabetic estrogenized mice at a lower inocula than that required for *C*. *glabrata* in diabetic estrogenized mice [6], possibly due to weaker adherence to the vaginal mucosa. The diabetic condition does not appear to contribute to the lack of responsiveness to *C*. *glabrata* as diabetic *C*. *albicans-*inoculated mice had similar immunopathogenic responses compared to non-diabetic counterparts.

*C*. *glabrata* has been shown to have a strong predilection for macrophages as an immune evasion strategy and postulated to attract macrophages for this purpose [25]. There are few resident macrophages at the vaginal mucosal and no demonstrable migration of macrophages during *C*. *albicans* infection [5, 26]. This appears to hold true for *C*. *glabrata* infections too. Hence, this survival mechanism would not seem to present at the vaginal mucosa.

*C*. *albicans* biofilm formation has been extensively characterized *in vitro* (reviewed in [27, 28]) and *in vivo* [20, 29], while *C*. *glabrata* biofilm formation has only been described on abiotic surfaces [30–32]. This study is the first to demonstrate *C*. *glabrata* does not form appreciable biofilm on vaginal mucosa. This conclusion is reasonable given that a mature *Candida* biofilm consists of hyphal elements encased in extracellular matrix [33]. Furthermore, *C*. *albicans* biofilm is dependent on the ability to undergo morphogenesis, as mutants defective in hyphal formation are also defective in biofilm formation *in vitro* and *in vivo* [20, 28, 34]. Therefore, the inability of *C*. *glabrata* to undergo morphogenesis likely precludes adequate biofilm maturation *in vivo*.

Synergistic effects on virulence have been demonstrated with *C*. *albicans* and a number of bacterial pathogens [35–38]. Furthermore, *C*. *albicans-C*. *glabrata* mixed species vaginal infections are relatively common [21] and studies using reconstituted human oral and vaginal epithelium have shown increased tissue damage and invasion during co-infection [39, 40]. In the present study, vaginal co-infections with *C*. *albicans* and *C*. *glabrata* did not result in amplified/synergistic pathogenesis. Fungal burdens were not enhanced during co-infection, PMNs were recruited to similar levels during co-infection compared to *C*. *albicans* monomicrobial infections, and similar levels of tissue damage (LDH) and inflammatory markers (data not shown) were observed compared to *C*. *albicans* mono-infection. As expected, *C*. *glabrata* mono-inoculated mice had significantly lower levels of all parameters compared to co-inoculated mice or *C*. *albicans* mono-inoculated mice. Fluorescent staining revealed that both *Candida* species are interspersed throughout the tissue under co-inoculated conditions, but there was little interaction or co-localization suggesting that both species persist relatively independently. Taken together, in contrast to *in vitro* models, there is no evidence for additive/synergistic effects of *C*. *glabrata* and *C*. *albicans* at the vaginal mucosa *in vivo*. The contrasting differences may in part be due to the *in vitro* tissue environment that favors/promotes interactions of the two pathogens. In the oral mucosa it has been postulated that *C*. *glabrata* can obtain nutrients from *C*. *albicans*-mediated tissue damage for stronger survival and invasion, possibly to a point of gaining entry into the bloodstream [41]. It is difficult to even speculate whether *C*. *glabrata* achieves enhanced fitness when present with *C*. *albicans* in the murine vaginal mucosa based on the 21-day observation period in the model. But suffice to say that there was no evidence of extra-vaginal *C*. *glabrata* migration and no recognizable changes in *C*. *glabrata* in the co-infection compared to the mono-infection based on the parameters investigated. Future studies can focus on more in-depth characteristics of *C*. *glabrata* in a co-infection environment.

In conclusion, a robust murine model of *C*. *glabrata* vaginitis has been established, which is characterized by high and consistent levels of *C*. *glabrata* colonization that are sustained over time (21 days post-inoculation). Using this model, we have demonstrated that *C*. *glabrata* does not elicit the hallmark *C*. *albicans-*associated immunopathology during vaginal colonization, and requires specific host parameters to persist on the tissue (hyperglycemia, estrogen). In fact, the infection/colonization in the absence of acute inflammation may be suggestive of the term ‘vaginosis’ rather than classical ‘vaginitis’ similar to the condition associated with bacterial vaginosis (BV). Importantly too, *C*. *glabrata* colonization does not synergize or enhance *C*. *albicans* pathogenesis in the experimental co-inoculated environment. This conclusion would support the clinical paradigm in which both species are commonly co-isolated, but duel detection is likely due to the frequency of *C*. *albicans* commensalism rather than a synergistic co-infection. Additionally, our data suggest that *C*. *glabrata* may not be responsible for the symptoms recorded during many cases in which *C*. *glabrata* is isolated, but may act as an innocent bystander while other underlying causes are responsible for the symptoms observed. Nevertheless, the difficulty associated with treating cases of VVC involving *C*. *glabrata* remains, but may be less of an issue if *C*. *glabrata* is not responsible for the symptomatic condition. This model will enable studies to be designed/performed to evaluate broader pathogenesis issues in mono- or co-infections, as well as provide a new tool for studying the *in vivo* efficacy of oral and topical antifungal agents against *C*. *glabrata* vaginal infections.
